# Supplementary material for: Functional outcome in home health: Do racial and ethnic minority patients with dementia fare worse?
Source: PLoS One. 2020 May 26;15(5):e0233650. doi: 10.1371/journal.pone.0233650 (PMC7250428; doi:10.1371/journal.pone.0233650)
Supplement: Attachment — Submitted filename: 1. Response to PLOS One reviewers comments (3-4-2020).docx [file pone.0233650.s001.docx]

Response to Reviewers

Manuscript ID: PONE-D-19-29178

February 21, 2020

Dear Dr. Heber,

Thank you for the review of our manuscript, " Functional Outcome in Home Health: Do minority patients with dementia fare worse?" (now entitled “Functional Outcome in Home Health: Do Racial and Ethnic Minority Patients with Dementia Fare Worse?”). We have thoroughly revised this manuscript and believe that we have adequately addressed the and Editor’s and reviewers’ concerns.

According to the Editor’s comments on additional journal requirements, we have made the following changes, including 1) ensuring meeting the PLOS ONE style requirements, 2) terminology (*‘Caucasian’ changed to ‘white’*), 3) revising the title, 4) statement about IRB approval (line 75-78), 5) full financial disclosure (*This study was conducted with the support of the following funders: Elaine C. Hubbard Center for Nursing Research on Aging Research Endowed Award (JW), Terry Family Research Endowed Award (JW), and the Valerie and Frank Furth Fund Award (JW). The funders had no role in study design, data collection and analysis, decision to publish, or preparation of the manuscript.*), and 6) decision on not making the data from the study publicly available and the rationale (*datasets contain confidential information of the patients and the collaborating home health agency that do not allow public data sharing*).

Below, we detailed our point-by-point response to the reviewers’ comments in the order they appeared in the decision letter. We uploaded two copies of the revised manuscript, including a marked copy with the revised text highlighted (‘Revised Manuscript with Track Changes’) and an unmarked, clean copy (‘Manuscript’).

**Reviewer Comments: #1**

This is the first submission of an article titled “Functional Outcomes in Home health: Do minority patients with dementia fare worse”. The authors are a team or researchers with expertise in nursing, biostatistics, geriatrics, and public health. Study purpose is to examine independent and interactive effects of dementia with racial/ethnic minority status on outcomes during home health admissions for those with Medicare. The study is a retrospective analysis of OASIS records from an agency in NY. The team proposes that the effects of home health on activity of daily living improvement is variable among racial/ethnic minorities and those with dementia. They hypothesized that during home health admission, minority patients with dementia will have less ADL improvement than non-Hispanic Caucasian patients. They used data from non-profit HH agencies to assess patient characteristics, socioeconomic status, living arrangement, social support, health status, and cognitive and physical function. The team reports that Hispanics and Caucasians without dementia had the greatest ADL improvement, while individuals who are African American or from other minorities with dementia had the least ADL improvement. I found these findings to be interesting – but do note some concerns regarding limited discussion of alternative viable hypotheses, consideration for education, consideration for severity of dementia, consideration for social economic status discrepancies.

**Response:** We have revised this manuscript as outlined in the detailed responses below.

1) In lines 326-330, we discussed the limitation of not having information on the severity of dementia in the OASIS dataset. In lines 80-83, we also categorized patients with dementia into five types, including vascular dementia, Alzheimer's disease, frontotemporal dementia, lewy body dementia, and dementia in other diseases classified elsewhere, and unspecified dementia. However, for two reasons, it was not feasible for us to control for the type of dementia in the analysis. First, among the 1156 patients in this sample who were identified with dementia, 414 had both a documented diagnosis of dementia and cognitive impairment in OASIS, 99 had only a dementia diagnosis, and 643 had only cognitive impairment symptoms. We did not have information about the type of dementia for the 643 patients who had cognitive impairment symptoms only, without a documented diagnosis. Second, even among the 513 (414+99) patients who had a documented diagnosis of dementia, 93% of them were documented with “dementia in other diseases classified elsewhere, and unspecified dementia” (absolute rates: 9.9% out of 10.7%), making it difficult to generate comparable groups by the type of dementia.
2) In lines 236-240, we discussed the limitation of not having information about education in OASIS and the need for such information in future studies.
3) In lines 217-223 and 253-255, we discussed the alternative hypothesis where racial and ethnic minority patients, due to their lower ADL status at baseline and differences in family and social support structures, may have more room for improvement in ADL while receiving HH services.
4) In lines 236-240, we discussed the impact of SES on health outcomes and the important role of SES to the racial/ethnic disparities in dementia outcomes.

Page 3: Inclusion criteria; I suggest participant design flow to show the method by which patient data were extracted. This will be thorough.

**Response:** In line 75, we added a participant flow chart (Figure 1). Please note that figure 1 in the previous submission is now named figure 2.

Page 3: incomplete sentence “This study was approved by the University of XXX Institutional review board. “ The university is not named here.

**Response:** In previous submission, we blinded the name of the university. In line 76, we included the name of the university.

Page 3: Diagnosis of dementia is via ICD-10 codes – but who diagnosed the dementia as well as the ADL status.

**Response:** In lines 63-64, 80-81 and 90-92 as well as 100-101, we explained that the ICD-10 codes in Medicare claims are documented by the physicians, and the ADL status in the OASIS is assessed by HH clinicians such as nurses and physical therapists during home health visits.

Major limitation: Cognitive function is not actually assessed, from my observation. It appears to be an opinion based on the data field entered in from the physician – but unknown how this was determined. Is this based on objective testing, patient family feedback, interviews, or simple behavioral observation?

**Response:** We appreciate the reviewer’s comment on the assessment of cognitive functioning, as it is an important aspect of this study. In line 63-64 and again lines 90-92 and lines 100-101, we noted that OASIS items are collected by HH clinicians, primarily nurses and physical/occupational therapists. In lines 90-92, we explained the source and timeline of cognitive functioning items in OASIS.

In the user manual of OASIS (<https://www.cms.gov/Medicare/Quality-Initiatives-Patient-Assessment-Instruments/HomeHealthQualityInits/Downloads/OASIS-C2-Guidance-Manual-6-17-16.pdf>) and based on the investigators’ clinical and research experience in home health care, the cognitive functioning items in OASIS are assessed in two ways, including 1) direct observation of the home health clinicians on the home visit; and 2) on the visit, having the patient and/or caregivers report to the home health clinician relevant cognitive function in the past 14 days. Prior research has shown that the cognitive functioning items in OASIS have satisfactory validity and reliability (lines 64-67; also included in prior submission).

Major limitation: Rationale for the hypothesis is unclear. The opposite pattern could be hypothesized – i.e., individuals with racial/ethnic diversity may have more improvement in ADL due to more cohesive families, perhaps. I encourage the team to clarify why racial/ethnic networks would be more vulnerable than individuals who are non-Hispanic/ Caucasian.

**Response:** In line 217-223, we discussed alternative hypothesis, where racial minority patients have more ADL limitation at baseline and thus more room for ADL improvement subsequently. In lines 253-255 we discussed how differences in the structure of family and social support, which may also contribute to the differences in ADL changes among the racial groups.

Major limitation is that education is not considered in this model.

**Response:** In lines 236-240, we discussed the limitation of not having information about education in OASIS and the need for such information in future studies.

Major limitation is that although the database assumes access to social economic status – I do not see SES mentioned in the table or the model outcome. It is mentioned once in the paper – and that is in the reference to the OASIS record. Can the authors please clarify if these data are able to be acquired?

**Response:** In lines 236-240, we discussed the limitation of SES measure in this study, particularly that we used dual eligibility as a proxy of SES. We emphasized the need for comprehensive data about socioeconomic status in future research.

Major limitation: The authors appear to assume the dementias are at the same level of severity. It is unknown if the African American minority patients were at a greater level of dementia – and were taken to the physician at a later time point due to cultural or family factors. This would explain the limited improvement. Also, type of dementia is not considered.

**Response:**

- In lines 326-330, we discussed the limitation of not having information on the severity of dementia in the OASIS dataset.
- In lines 80-83, we also categorized patients with a diagnosis of dementia into five types, including vascular dementia, Alzheimer's disease, frontotemporal dementia, lewy body dementia, and dementia in other diseases classified elsewhere, and unspecified dementia.

**Reviewer Comments: #2**

This manuscript addresses an important topic with several key implications for the management of dementia in those receiving home care. It highlights racial disparity in outcomes of older adults receiving home care. The authors have used a rich data source that was well-described for the analysis. However, I have some reservations about the study’s conclusions because of the analysis. These are listed below

**Response:** Thank you for acknowledging our work. We have revised this manuscript as outlined in the detailed responses below.

1. The authors used multiple linear regression for the analysis without examining whether the assumptions of linear regression are satisfied in the data. More specifically, the assumption of normal distributions was not examined. The authors should present the distribution of the change scores and the corresponding regression residuals.

**Response:** We appreciated the reviewer’s comment. We tested and found that the assumptions of linear regression were met. Specifically, normality assumption of ADL changes generated by OASIS items – same method of calculating ADL score change as we did in this paper - has been accepted empirically (see doi: 10.1016/j.archger.2011.07.018). Further, central limit theorem holds approximate normality of sampling distribution as long as the distribution has legitimate expectation and variance under large sample size. Due to the above reasons, we are comfortable to use the linear regression model in this analysis.

Per reviewer’s interest we provided the residual plot of the fitted model (below), which showed relatively evenly distributed residuals among the subgroups, with a few outliers in the white subgroup probably due to very large sample size.

The notation for group numbers is as such: group 1= white without dementia; group 2= white with dementia; group 3 = black without dementia; group 4 = black with dementia; group 5 = Hispanic without dementia; group 6 = Hispanic with dementia; group 7 = others (Asians) without dementia; and group 8 = others (Asians) with dementia.

2. A distribution of the change scores should be presented along with an estimate of the effect size

**Response:**

1) Due to lack of space in the manuscript, we provided the histogram in the response letter. Again the normality assumption can be accepted according to the central limit theorem.
2) Effect size estimates of the effect of having dementia and racial/ethnic minority status on the ADL outcome were presented in Table 2 (regression coefficient β). In addition, we provided the full regression model solution table of the final model with interaction between dementia and racial/ethnic status. We did not include standard error and t into the presented table as they were often not of interest to the readers.
3) The histogram below showed an approximately bell-curve distribution of ADL changes score, indicating appropriateness of the normality assumption, as presented below.

The final model with effect size of each variable:

| ADL score change (score negative: improvement; positive: increase) | Coef. | Std. Err. | t | P>t | 95% Confidence Interval | | Effect size (Coef/Std.Err.) |
| --- | --- | --- | --- | --- | --- | --- | --- |
| Race/ethnicity and dementia interaction (reference: White without dementia) | | | | | | | |
| - White, dementia | 0.513 | 0.054 | 9.570 | 0.000 | 0.408 | 0.619 | 9.572 |
| - African American, no dementia | 0.207 | 0.074 | 2.800 | 0.005 | 0.062 | 0.351 | 2.805 |
| - African American, dementia | 1.077 | 0.139 | 7.770 | 0.000 | 0.805 | 1.349 | 7.768 |
| - Hispanic, no dementia | 0.026 | 0.183 | 0.140 | 0.887 | -0.332 | 0.384 | 0.142 |
| - Hispanic, dementia | 0.922 | 0.279 | 3.300 | 0.001 | 0.375 | 1.469 | 3.305 |
| - Asian, no dementia | 0.018 | 0.207 | 0.090 | 0.929 | -0.387 | 0.424 | 0.089 |
| - Asian, dementia | 1.469 | 0.336 | 4.370 | 0.000 | 0.811 | 2.128 | 4.373 |
| Medicare-Medicaid Dual eligibility (reference: No) | 0.175 | 0.136 | 1.290 | 0.198 | -0.091 | 0.442 | 1.289 |
| Age | 0.019 | 0.002 | 7.760 | 0.000 | 0.014 | 0.023 | 7.763 |
| Female (reference: male) | -0.014 | 0.042 | -0.330 | 0.739 | -0.095 | 0.068 | -0.334 |
| Married (reference: not married) | -0.090 | 0.047 | -1.900 | 0.058 | -0.182 | 0.003 | -1.899 |
| Living arrangement (reference: living with others at home) | | | | | | | |
| - Live alone | -0.335 | 0.054 | -6.180 | 0.000 | -0.441 | -0.229 | -6.183 |
| - Assisted living facilities | 0.113 | 0.073 | 1.560 | 0.118 | -0.029 | 0.255 | 1.563 |
| Referral source (reference: after hospitalization) |  |  |  |  |  |  |  |
| - After post-acute care | 0.147 | 0.061 | 2.400 | 0.016 | 0.027 | 0.267 | 2.399 |
| - Community | 0.683 | 0.049 | 14.030 | 0.000 | 0.588 | 0.779 | 14.026 |
| Number of diagnoses | 0.014 | 0.007 | 2.040 | 0.042 | 0.001 | 0.027 | 2.036 |
| Number of medications | -0.001 | 0.004 | -0.340 | 0.732 | -0.009 | 0.006 | -0.342 |
| Heart failure (Yes/No) | 0.123 | 0.055 | 2.240 | 0.025 | 0.015 | 0.230 | 2.240 |
| COPD (Yes/No) | -0.119 | 0.056 | -2.120 | 0.034 | -0.229 | -0.009 | -2.118 |
| Diabetes without complications (Yes/No) | -0.008 | 0.053 | -0.160 | 0.875 | -0.112 | 0.095 | -0.158 |
| Diabetes with complications (Yes/No) | 0.071 | 0.066 | 1.080 | 0.280 | -0.058 | 0.201 | 1.081 |
| Obesity (Yes/No) | -0.014 | 0.048 | -0.280 | 0.776 | -0.109 | 0.081 | -0.285 |
| Smoking (current or past; Yes/No) | -0.058 | 0.054 | -1.070 | 0.284 | -0.164 | 0.048 | -1.072 |
| Daily interfering pain (Yes/No) | -0.300 | 0.045 | -6.730 | 0.000 | -0.388 | -0.213 | -6.729 |
| Depressive symptoms (Yes/No) | 0.162 | 0.041 | 3.940 | 0.000 | 0.081 | 0.243 | 3.945 |
| Receiving daily caregiver assistance | -0.046 | 0.059 | -0.780 | 0.433 | -0.161 | 0.069 | -0.784 |
| Baseline functional limitation | -0.395 | 0.018 | -22.050 | 0.000 | -0.430 | -0.360 | -22.051 |
| Service use during the index home health admission |  |  |  |  |  |  |  |
| - Total hours of skilled nursing | 0.006 | 0.004 | 1.620 | 0.105 | -0.001 | 0.014 | 1.620 |
| - Total hours of physical therapy | -0.030 | 0.004 | -7.500 | 0.000 | -0.037 | -0.022 | -7.504 |
| - Total hours of occupational therapy | 0.000 | 0.000 | -0.990 | 0.322 | -0.001 | 0.000 | -0.991 |
| - Total hours of aide assistance (homemaking) | 0.002 | 0.002 | 1.010 | 0.311 | -0.001 | 0.004 | 1.014 |
| - Total hours of social work | 0.169 | 0.052 | 3.270 | 0.001 | 0.068 | 0.271 | 3.270 |

3. I am surprised that the authors did not examine the direct effect of ethnicity and dementia on functional outcomes but only reported the ethnicity-dementia interactive effects. The authors should revise the analysis to include ethnicity and dementia as main effects in addition to the interaction effects.

**Response:** In lines 180-183, we reported results of the independent effects of minority and dementia status on the outcomes (also included in prior submission but highlighted here). We further presented the independent and interactive effects of minority and dementia status as shown in Table 2.
